# Trends in Research Related to Premenstrual Syndrome and Premenstrual Dysphoric Disorder From 1945 to 2018: A Bibliometric Analysis

**DOI:** 10.3389/fpubh.2021.596128

**Published:** 2021-04-21

**Authors:** Mingzhou Gao, Dongmei Gao, Hui Sun, Xunshu Cheng, Li An, Mingqi Qiao

**Affiliations:** ^1^College of Traditional Chinese Medicine, Shandong University of Traditional Chinese Medicine, Jinan, China; ^2^School of Pharmaceutical Sciences, South-Central University for Nationalities, Wuhan, China; ^3^Traditional Chinese Medicine Department, Jinan Central Hospital, Jinan, China

**Keywords:** PMS/PMDD, CiteSpace, menstrual cycle, trend, bibliometric analysis

## Abstract

**Background:** The global incidence of premenstrual syndrome (PMS) and premenstrual dysphoric disorder (PMDD) is increasing, with increasing suicide reports. However, the bibliometric analysis of global research on PMS and PMDD is rare. We aimed to evaluate the global scientific output of research on PMS and PMDD and to explore their research hotspots and frontiers from 1945 to 2018 using a bibliometric analysis methodology.

**Methods:** Articles with research on PMS and PMDD between 1945 and 2018 were retrieved from the Web of Science Core Collection (WoSCC). We used the bibliometric method, CiteSpace V and VOSviewer to analyze publication years, journals, countries, institutions, authors, research hotspots, and trends. We plotted the reference co-citation network, and we used keywords to analyze the research hotspots and trends.

**Results:** We identified 2,833 publications on PMS and PMDD research from 1945 to 2018, and the annual publication number increased with time, with fluctuations. Psychoneuroendocrinology published the highest number of articles. The USA ranked the highest among the countries with the most publications, and the leading institute was UNIV PENN. Keyword and reference analysis indicated that the menstrual cycle, depression and ovarian hormones were the research hotspots, whereas prevalence, systematic review, anxiety and depression and young women were the research frontiers.

**Conclusions:** We depicted overall research on PMS and PMDD by a bibliometric analysis methodology. Prevalence and impact in young women, systematic review evaluations of risk factors, and the association of anxiety and depression with menstrual cycle phases are the latest research frontiers that will pioneer the direction of research in the next few years.

## Introduction

Premenstrual syndrome (PMS), a common cyclical and recurrent disorder of the reproductive female population, is characterized by psychological and somatic symptoms that consistently occur during the luteal phase of the menstrual cycle (1). Women with more severe affective symptoms are classified as having premenstrual dysphoric disorder (PMDD), which is hereby designated as a depressive disorder in the DSM-5 (2, 3). Studies have shown that 75% of women of reproductive age are affected by disturbing premenstrual symptoms (4) and 5–8% of women thus suffer from severe PMS (2), and PMDD affects 3–8% of menstruating women (5). Diagnostic criteria for PMDD have been revised and updated, referring to newly discovered results from the Diagnostic and Statistical Manual of Mental Disorders IV (DSM-4) (6) to the Diagnostic and Statistical Manual of Mental Disorders V (DSM-5) (7). In clinical diagnosis, the DSM-5 requires at least five specified symptoms for PMDD, while the ICD-10 requires only one distressing symptom for diagnosis of PMS (8).

Clinically, depression, anxiety, and irritability are seen as the three most studied symptoms of PMDD (9). And abdominal bloating, cramps or abdominal pain, irritability, and joint/muscle/back pains were the most prevalent symptoms in patients from France, Germany, Hungary, Italy, Spain, UK, Brazil, Mexico, Hong Kong, Pakistan, and Thailand (10). Recently, anger appears to be an important problem that makes life more difficult for subjects with PMDD (11). And there is a strong, independent association between PMS/PMDD and trait anger among a representative sample of female suicide attempters (12). Besides, recent evidence from studies indicates that sex steroids (progesterone, allopregnanolone, and estrogen) and central neurotransmitters (serotonin, gamma-aminobutyric acid, glutamate and beta endorphins) maybe core mechanism (13–16). Psychological factors including perceived stress, tobacco consumption (17), neuroticism and coping strategies (negative cognitive styles) are strongly related to PMS/PMDD (18). It has been suggested that PMDD is a manifestation of the underlying depressive disorder which is associated with the inability to regulate emotions in an adaptive manner (19).

As research in PMS/PMDD advances rapidly, there still have been no attempts to systematically analyze the data on publications with so many scholarly papers published in journals in PMS/PMDD research over the past decade. Luckily, a bibliometric analysis methodology is widely used to assess trends in research activities as a quantitative analysis combining mathematical and statistical methods (20). Bibliometric analysis was utilized to detect the knowledge structure and emerging trends by quantitative analysis (21). In this study, CiteSpace (22) and VOSviewer (23) were applied to conduct a bibliometric analysis of related references derived from the Science Citation Index-Expanded (SCI-E) of the Web of Science database from 1945 to 2018. And this comprehensive bibliometric analysis that we will make aims to provide an overview of the status and to keep abreast of emerging trends and critical turns of the development of global research of PMS/PMDD.

## Methods

### Data Source and Search Strategy

Literature retrieval was performed online through the SCI-E of the Web of Science Core Collection (WoSCC) on September 23, 2018. All searches were performed within the same day to avoid the bias caused by the daily database updates. The search queries are listed in Table 1.

**Table 1:** Search queries for premenstrual syndrome and premenstrual dysphoric disorder.

**Mark**	**Number**	**Queries**
# 1	3064	TS=(Premenstrual Syndrome) OR TS=(Premenstrual Syndromes) OR TS=(Syndrome, Premenstrual) OR TS=(Syndromes, Premenstrual) OR TS=(Premenstrual Tension) OR TS=(Premenstrual Tensions) OR TS=(Tension, Premenstrual) OR TS=(Tensions, Premenstrual) Index =SCI-EXPANDED, CCR-EXPANDED, IC Time Span = All years
# 2	1583	TS=(Premenstrual Dysphoric Disorder) OR TS=(Disorder, Premenstrual Dysphoric) OR TS=(Dysphoric Disorder, Premenstrual) OR TS=(Premenstrual Dysphoric Syndrome) OR TS=(Syndrome, Premenstrual Dysphoric) Index =SCI-EXPANDED, CCR-EXPANDED, IC Time Span = All years
# 3	3852	#2 OR #1 Index =SCI-EXPANDED, CCR-EXPANDED, IC Time Span = All years
# 4	2833	#3 Refining basis: [Exclusion] Document type: (PROCEEDINGS PAPER OR NEWS ITEM OR REPRINT OR EDITORIAL MATERIAL OR CORRECTION OR ABSTRACT OF PUBLISHED ITEM OR MEETING ABSTRACT OR NOTE OR BOOK CHAPTER OR CORRECTION ADDITION OR LETTER OR BOOK REVIEW OR DISCUSSION) AND language: (ENGLISH) Time Span: all year. Index: SCI-EXPANDED, SSCI, CCR-EXPANDED, IC.

### Inclusion and Exclusion Criteria

**Figure 1:**
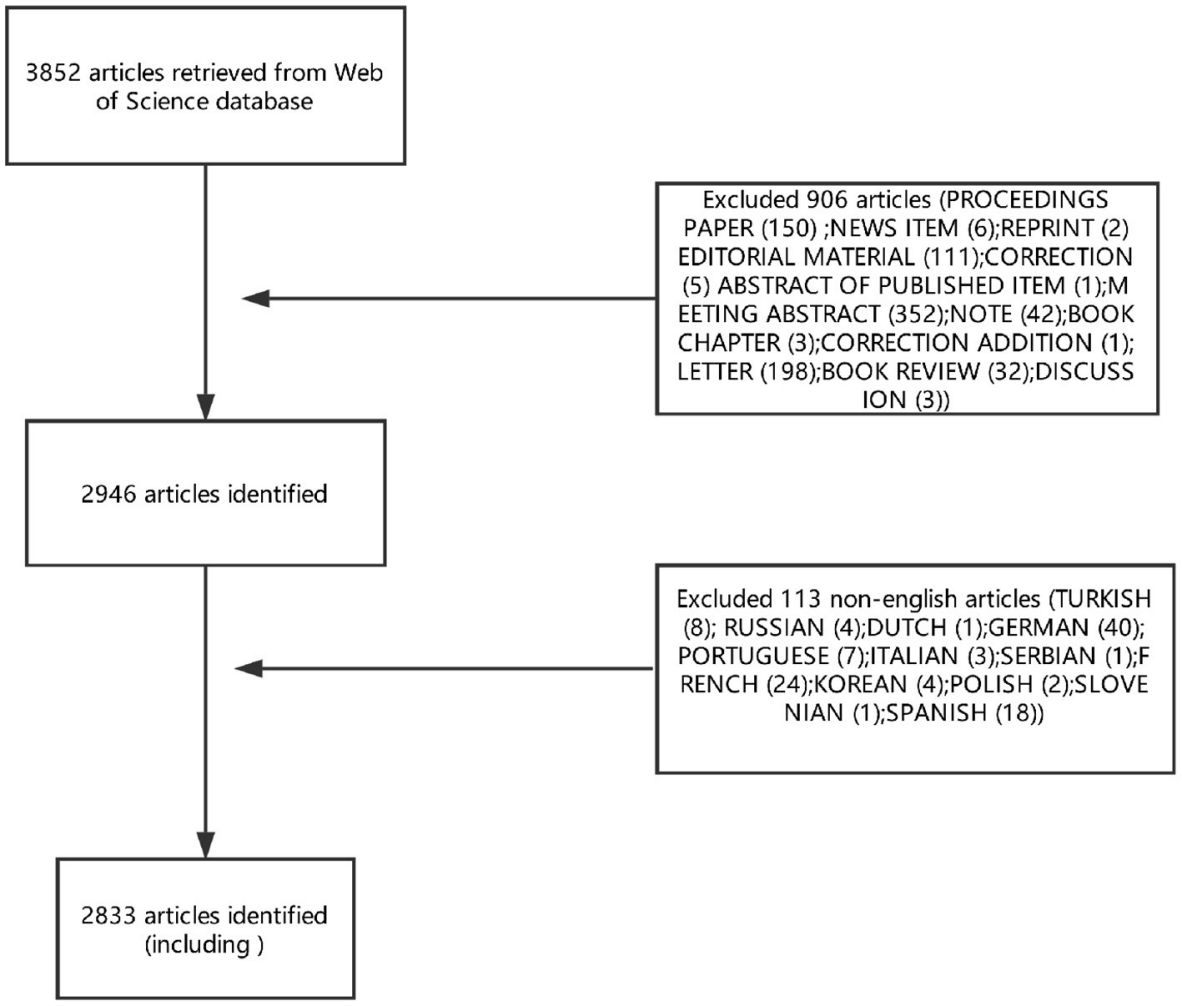
Flow diagram of study selection of PMS/PMDD research.

Articles or reviews that met the following criteria were included: (1) articles published between 1945 and September 23, 2018; (2) articles indexed in the WoSCC; (3) articles on PMS/PMDD research, including original research and reviews; and (4) articles with basic information. The following documents were excluded: (1) meeting abstracts, proceedings, corrected articles, and repeated articles; (2) unpublished documents without enough information for further analysis; and (3) non-English publications. The screening and review strategy is illustrated in Figure 1.

### Data Collection

Raw data from the WoSCC were initially downloaded and verified by two authors (Mingzhou Gao and Hui Sun) independently. The data were then imported into Excel 2019, VOSviewer and CiteSpace V and systematically analyzed.

### Statistical Methods

A descriptive analysis was used to present the characteristics of the included studies by publication years, countries, journals, and authors. Then, we used CiteSpace V (version 5.3. R4) to construct knowledge maps. In addition, we also used VOSviewer software (version 1.6.9) for better network visualizations in some cases. Finally, we also applied burst detection to investigate the growth rate of citations or keywords with CiteSpace V.

## Results

### Annual Publication Outputs

**Figure 2:**
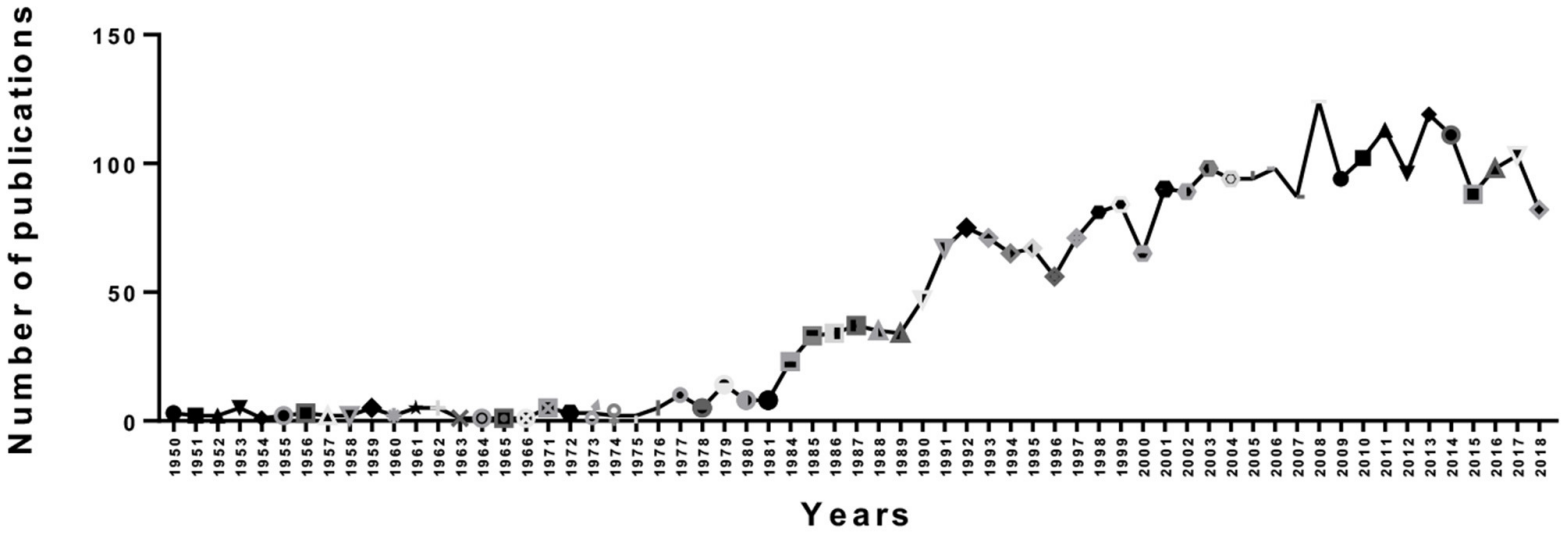
The number of annual publications on PMS/PMDD research from 1950 to 2018.

As showed in Figure 2, we counted the number of publications each year. Overall, there was an upward trend in publications from 1950 to 2008, but there were fluctuations in some years. However, we can see that the number of papers published in a single year reached a maximum of 124 in 2008. Then, there was a gradual downward trend from 2008 to 2018. Moreover, we can see that the trend in output was not stable; since 2000, there were fluctuations in 2001, 2007, 2009, 2012, 2016, and 2017. This finding may be due to the publication cycle. On the one hand, an upward trend indicates that PMS/PMDD research is becoming a hotspot, but a download trendency indicates that PMS/PMDD research attention tends to be flat.

### Distribution of Journals and Co-cited Journals

In total, 295 academic journals have published articles on PMS/PMDD. According to the Journal Citation Reports (JCR) 2017 standards, the top 15 journals contributing to PMS/PMDD are shown in Table 2. Psychneuroendocrinology [impact factor (IF) 2017 = 4.731] published the most papers (79 publications, 2.789%), followed by the Journal of Psychosomatic Obstetrics and Gynecology (IF 2017 = 1.900, 61 publications; 2.153%), the American Journal of Obstetrics and Gynecology (IF 2017= 5.732, 50 publications; 1.765%) and Gynecological Endocrinology (IF 2017 = 1.453, 49 publications; 1.730%).

**Table 2:** The top 15 journals with publications on PMS/PMDD research from 1945 to 2017.

**Journal**	**Count**	**% of 2,833**	**IF 2017**
Psychoneuroendocrinology	79	2.789	4.731
Journal of Psychosomatic Obstetrics and Gynecology	61	2.153	1.900
American Journal of Obstetrics and Gynecology	50	1.765	5.732
Gynecological Endocrinology	49	1.73	1.453
Journal of Affective Disorders	49	1.73	3.786
Obstetrics and Gynecology	47	1.659	4.982
Archives of Women's Mental Health	42	1.483	2.565
Journal of Reproductive Medicine	38	1.341	0.452
Journal of Women's Health	38	1.341	2.097
Journal of Psychosomatic Research	37	1.306	2.947
Journal of Clinical Psychiatry	35	1.235	4.247
Biological Psychiatry	31	1.094	11.982
Acta Obstetricia et Gynecologica Scandinavica	29	1.024	2.649
Psychosomatic Medicine	29	1.024	3.810

In addition, Biological Psychiatry (IF 2018 = 11.982) had an IF higher than 10, and Gynecology (IF 2018 = 5.732) had an IF between 10 and five. Five journals, Obstetrics and Gynecology (IF 2018 = 4.982), Psychoneuroendocrinology (IF 2018 = 4.731), the Journal of Clinical Psychiatry (IF 2018 = 4.247), Psychosomatic Medicine (IF 2018 = 3.810), and the Journal of Affective Disorders (IF 2018 = 3.786), had an IF between five and three.

**Figure 3:**
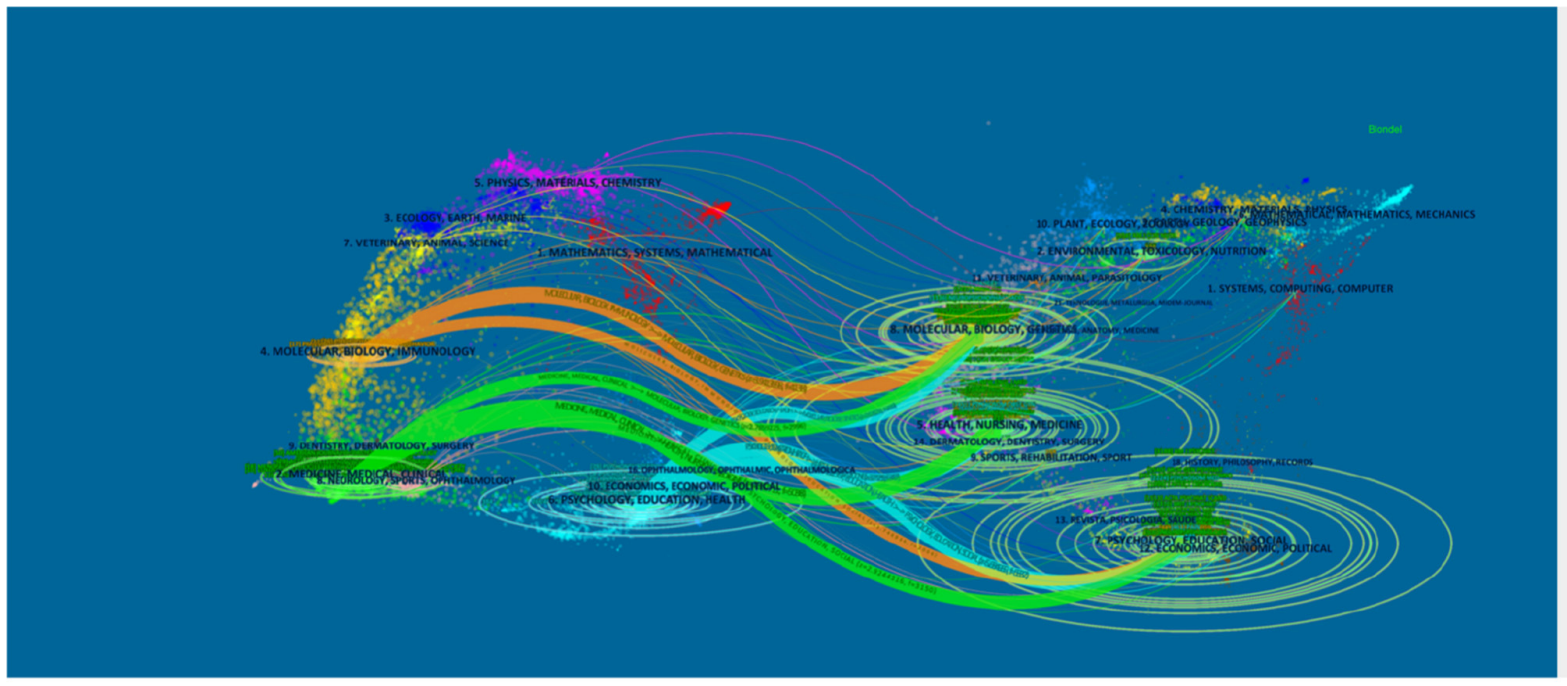
The dual-map overlay of journals related to PMS/PMDD research. The left and right sides corresponded to the citing and cited journals maps, respectively. The labels represented the disciplines covered by the journal. The lines on the map started from the left and ended on the right, representing the citation links.

We used CiteSpace's dual-map overlay function to construct a citation dual-map to visualize a more comprehensive view of the citation state of PMS/PMDD. As showed in Figure 3, dual-map overlays could show the interactions of journals. And the left and right sides correspond to the citing and cited journal maps, respectively. The labels represent the disciplines covered by the journal. The lines on the map starting from the left and ending at the right represent the citation links.

There is eight citation paths in the dual-map overlays. The upward yellow path shows that papers published in immunology/biology journals mostly cited journals in the area of biology/genetics. The downward yellow path shows that papers published in immunology/biology journals mostly cited journals in education/social areas. The upward green path shows that papers published in medicine/medical/clinical journals mostly cited journals in the area of molecular biology/biology/genetics. The middle green path shows that papers published in medical/clinical journals partially cited journals in the health/nursing area. The bottom green path shows that papers published in medicine/medical/clinical journals partially cited journals in the psychology/education/social area. The upward blue path shows that papers published in psychology/education/health journals mostly cited journals in the molecular biology/biology/genetics area. The middle blue path shows that papers published in psychology/education/health journals partially cited journals in the health/nursing/medicine area. The bottom blue path shows that papers published in psychology/education/health journals partially cited journals in the psychology/education/social area (Figure 3).

### Distribution of Countries/Regions and Institutes

**Figure 4:**
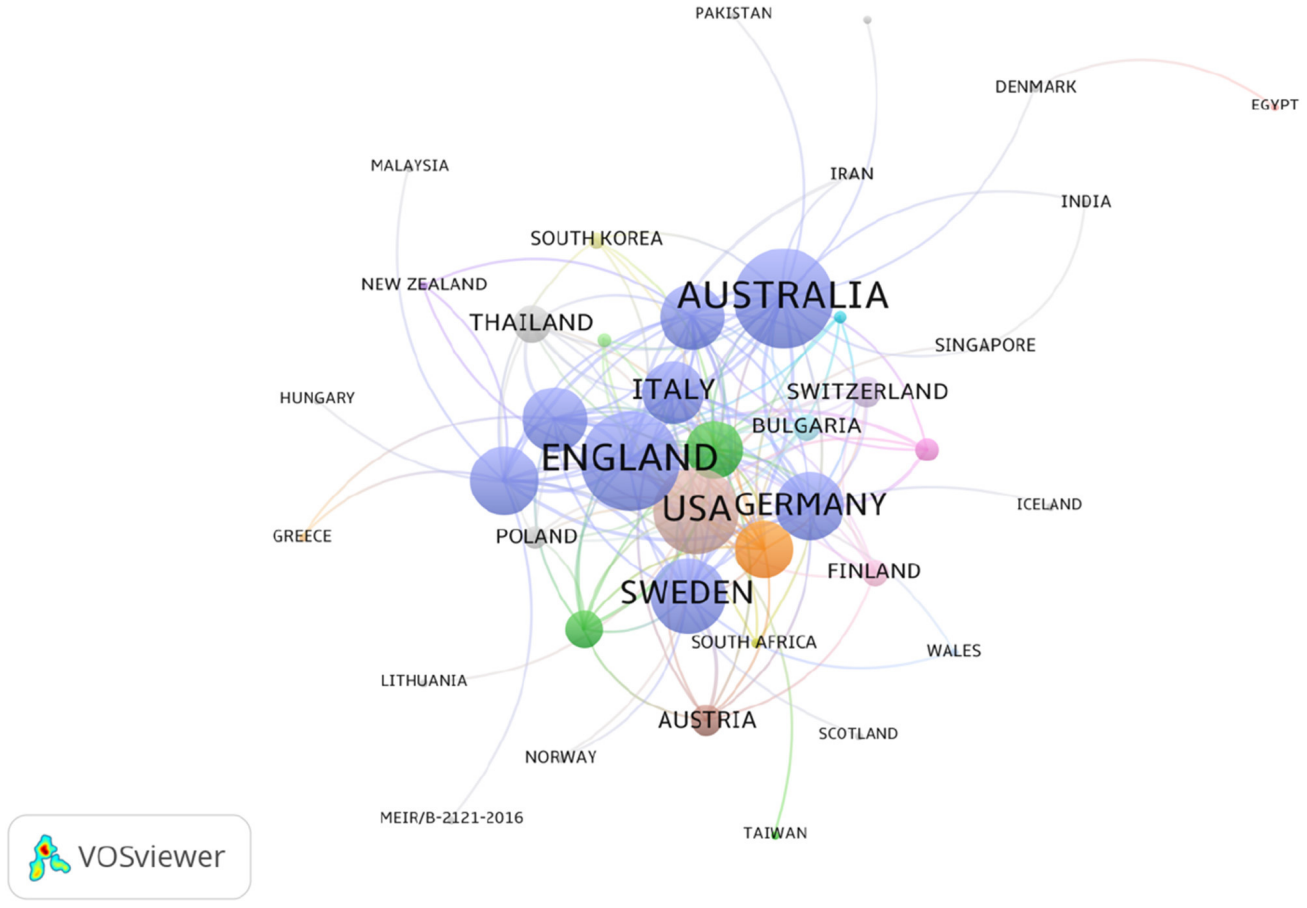
Map of countries/regions that published articles on PMS/PMDD during 1945–2018.

The 2,833 publications on PMS/PMDD were contributed by 76 countries/regions. There was extensive collaboration between countries/regions (Figure 4). As showed in Figure 4, colors showed different research directions. The larger nodes represented the more influential countries in this field. In relation to the top 10 countries that contributed PMS/PMDD research, the USA had the largest number of publications (1242), pursued by England (274), Sweden (2267), and Canada (940) (Table 3). Among the top 10 countries/regions in PMS/PMDD research, there is two Asian countries, China and Japan. China is the sole country from the developing world to be in the top 10 countries that contributed PMS/PMDD research, showing its vast progress in life science over the past decade.

**Table 3:** The top 10 country/regions and institutions of PMS/PMDD research.

**Rank**	**Country/region**	**Count**	**Institute**	**Count**
1	USA	1,242	University of Pennsylvania	71
2	England	274	National Institute of Mental Health	70
3	Sweden	194	University of California, Los Angeles	70
4	Canada	174	Umea University	60
5	Australia	140	University of North Carolina	57
6	Italy	125	Harvard University	51
7	Germany	100	Yale University	51
8	Netherlands	77	McMaster University	49
9	Japan	74	University of California, San Diego	47
10	Peoples R China	62	Suny Buffalo	44

**Figure 5:**
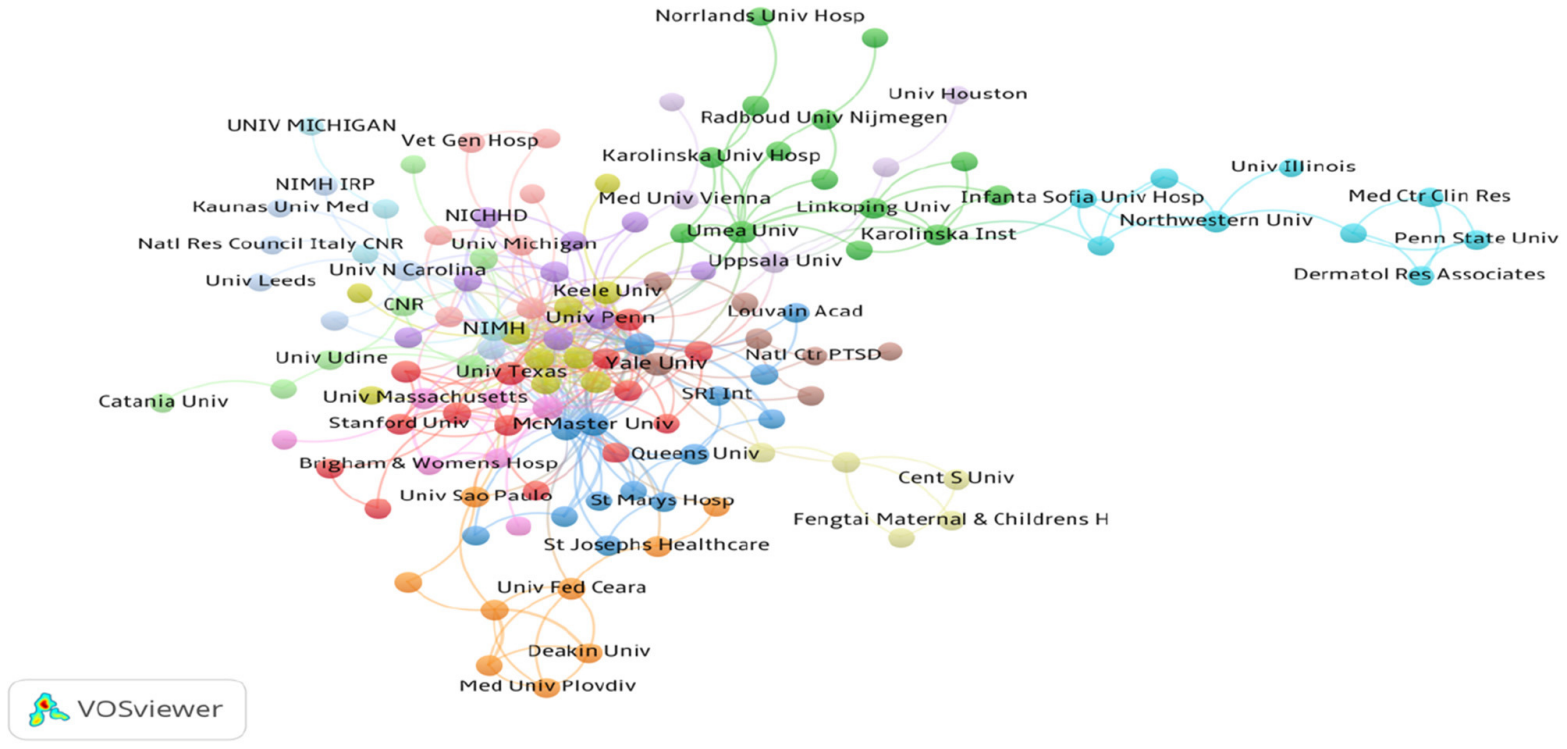
Map of institutions that published articles on PMS/PMDD during 1945–2018.

Over 1,700 institutions contributed to the publications on PMS/PMDD. Compared with countries, there was very little cooperation between the institutions (Figure 5). The lines between nodes represent the cooperative relationships among institutes. The length and thickness of the lines is the degree of cooperation among countries. The top 10 institutions contributed to 570 articles, which accounted for 20.12% of the total number of publications. The University of Pennsylvania led the first research echelon, followed by the National Institute of Mental Health H, University of California, Los Angeles, Umea University, University of North Carolina, and Harvard University (Table 3).

### Analysis of Author and Co-cited Author

**Figure 6:**
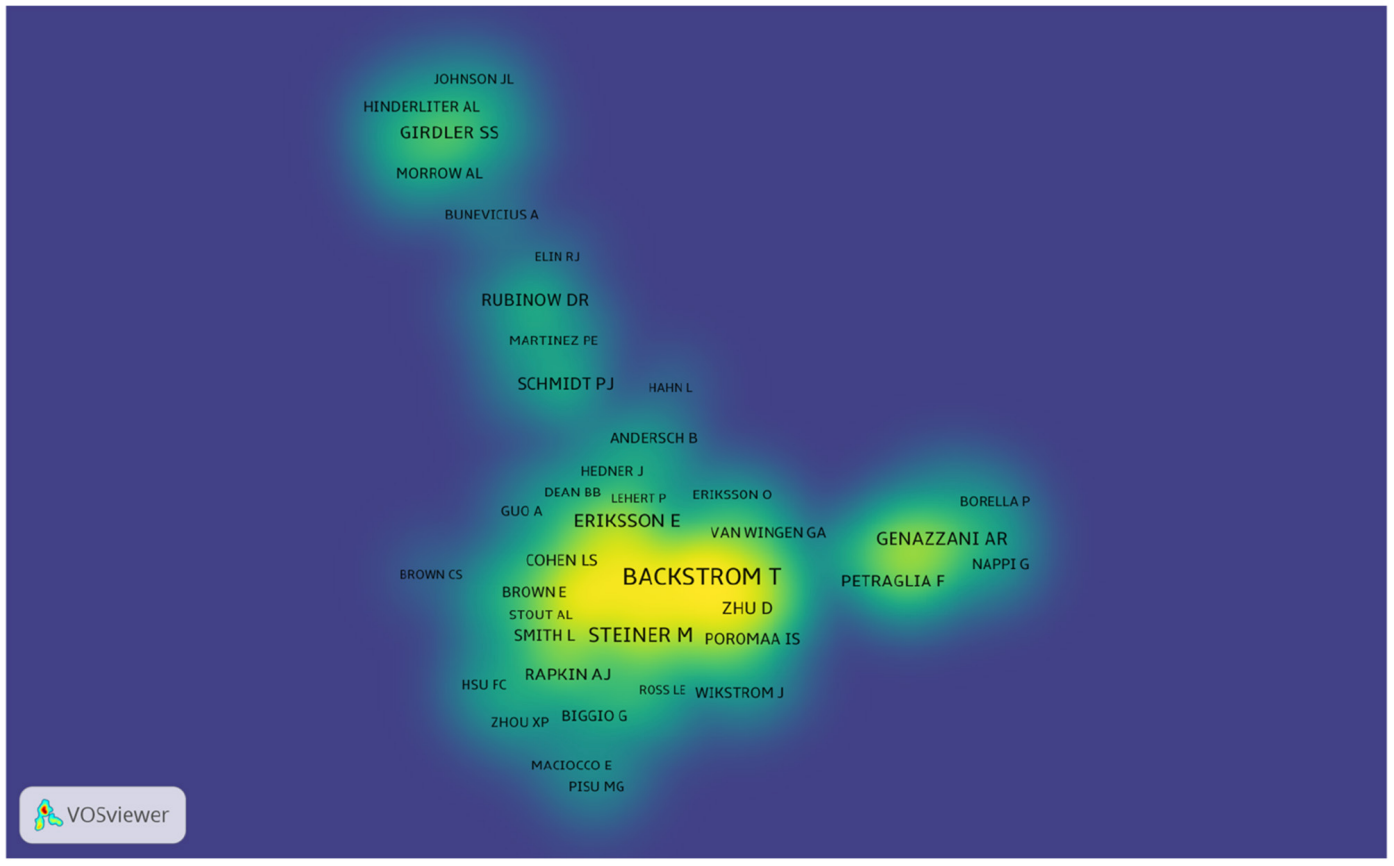
Map of authors that published articles on PMS/PMDD during 1945–2018.

More than 6,640 authors contributed to the total number of publications. The cooperation between authors is provided in a network map (Figure 6). The size of the nodes is equal to the number of citations. For authors who had the most publications, Backstrom T ranked the first (105 publications), followed by Rubinow DR (59 publications), Freeman EW (49 publications), and Schmidt PJ (45 publications) (Figure 6 and Table 3).

**Figure 7:**
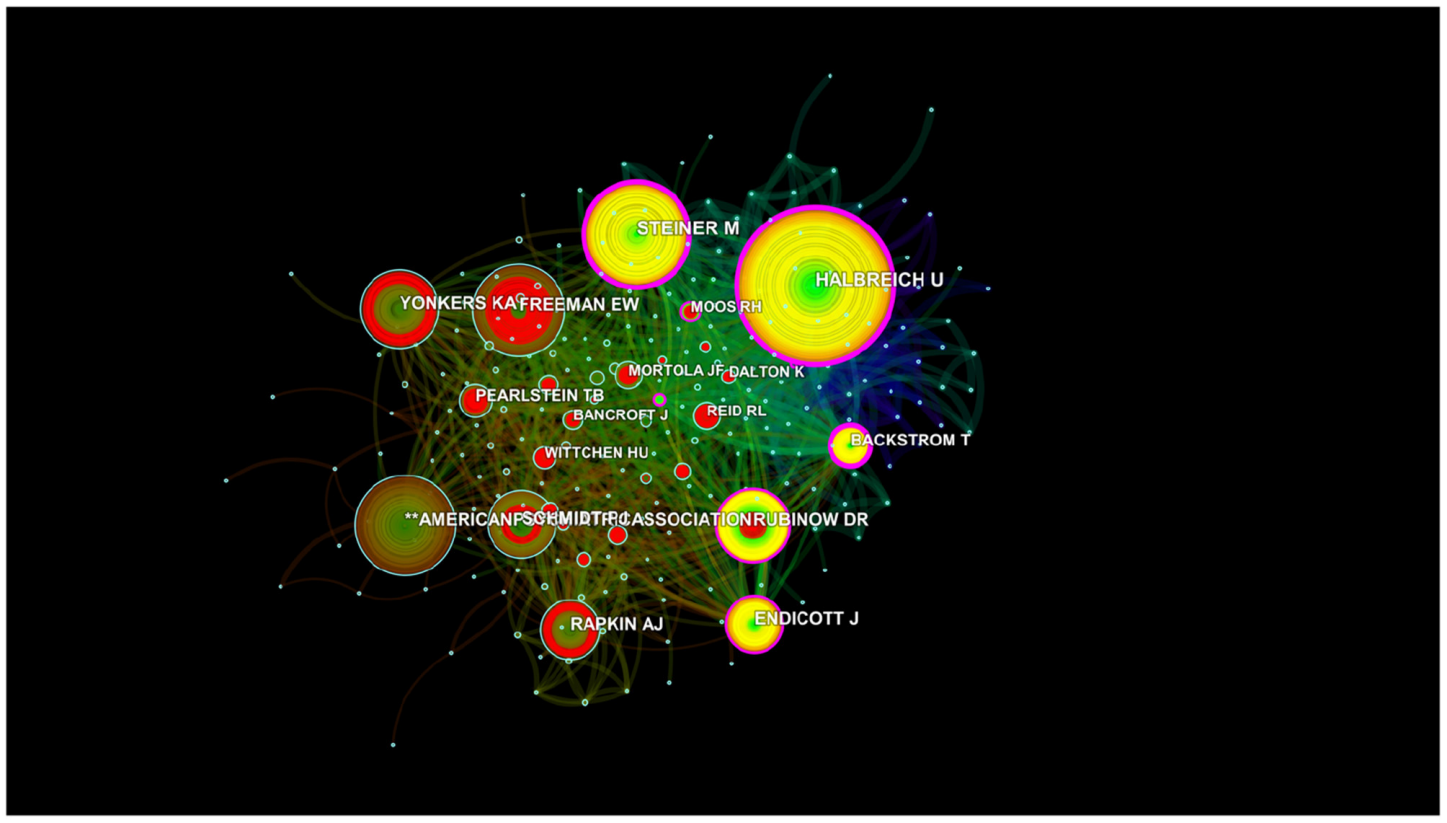
Map of co-cited authors that published articles on PMS/PMDD during 1945–2018.

Author citations tend to estimate the scientific relevance of publications. The top ranked author by citation count is Halbreich U (1978), with a citation count of 890. The second one is Steiner M (1980), with a citation count of 699. The third is ^**^the American Psychiatric Association (1986), with a citation count of 616. The 4th is Freeman EW (1988), with a citation count of 603. The 5th is Rubinow DR (1985), with a citation count of 492. The 6th is Yonkers KA (1996), with a citation count of 491. The 7th is Endicott J (1984), with a citation count of 467. The 8th is Schmidt PJ (1992), with a citation count of 463. The 9th is Rapkin AJ (1990), with a citation count of 428. The 10th is Backstrom T (1978), with a citation count of 393 (Table 4 and Figure 7).

**Table 4:** The top 10 active authors with publications on PMS/PMDD research from 1995 to 2018.

**Rank**	**Author**	**Count**	**Co-cited author**	**Count**
1	Backstrom T	105	Halbreich U	890
2	Rubinow DR	59	Steiner M	699
3	Freeman EW	49	American Psychiatric Association	616
4	Schmidt PJ	45	Freeman EW	603
5	Halbreich U	43	Rubinow DR	492
6	Steiner M	42	Yonkers KA	491
7	Eriksson E	33	Endicott J	467
8	Nyberg S	32	Schmidt PJ	463
9	Girdler SS	31	Rapkin AJ	428
10	Yonkers KA	31	Backstrom T	393

### Analysis of Co-cited References

**Figure 8:**
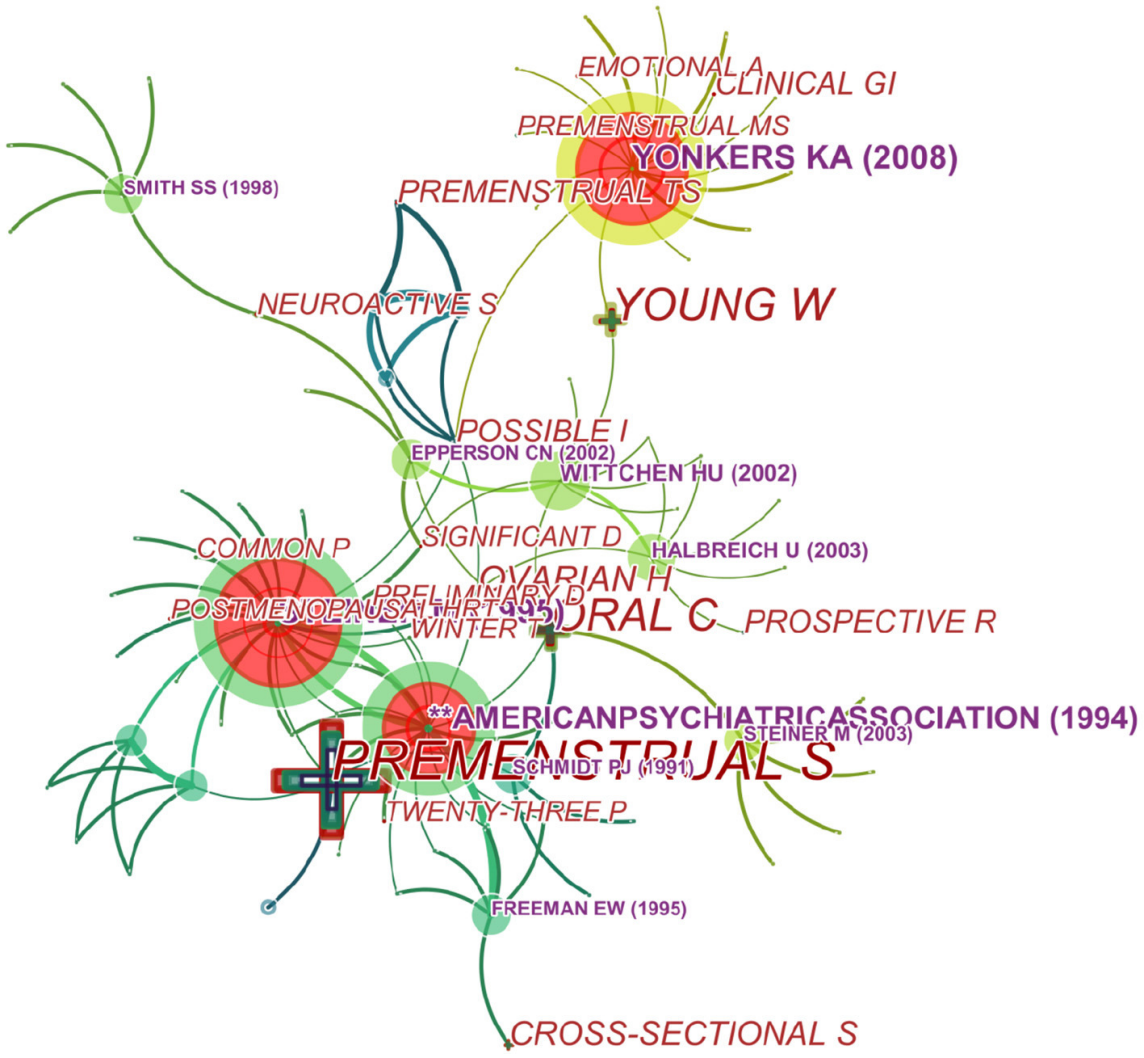
Map of co-cited references that published articles on PMS/PMDD during 1945–2018.

We generated a cited reference co-citation map by selecting the top 10 articles per year and mapping them in 485 nodes and 241 links (Figure 8). An analysis in terms of co-citation counts (Table 5 and Figure 8) revealed that the data on this topic over the past years were generally in the form of randomized trials, comparisons of diagnostic criteria (PSST) (28), pathogenesis (GABAA Receptor) and so on. For stance, Su et al. (24) performed a double-blind, placebo-controlled, crossover trial of fluoxetine in 17 women with PMDD and they confirmed that fluoxetine is an effective treatment of PMDD with highest frequency. And pathogenesis was studied focusing on endogenous steroid, GABAA Receptor, progesterone, and gamma-aminobutyric acid (29–31).

**Table 5:** The top 10 co-cited references on PMS/PMDD during 1945–2018.

**Rank**	**Freq**	**Author**	**Year**	**Source**	**Title**
1	21	Steiner M (24)	1995	New Engl J Med	Fluoxetine in the Treatment of Premenstrual Dysphoria
2	19	Yonkers KA (2)	2008	Lancet	Premenstrual Syndrome
3	17	American Psychiatric Association (6)	1994	Diagn Stat Man Ment	Diagnostic and Statistical Manual of Mental Disorders (4th ed.)
4	9	Reid RL (25)	1981	Am J Obstet Gynecol	Premenstrual Syndrome
5	8	Steiner M (26)	1980	Acta Psychiat Scand	Treatment of Premenstrual Tension with Lithium Carbonate. A Pilot Study
6	7	Wittchen HU (27)	2002	Psychol Med	Prevalence, Incidence and Stability of Premenstrual Dysphoric Disorder in the Community.
7	5	Steiner M (28)	2003	Arch Womens Ment Health	The Premenstrual Symptoms Screening Tool (PSST) for Clinicians
8	5	Smith SS (29)	1998	Nature	GABAA Receptor α4 Subunit Suppression Prevents withdrawal Properties of an Endogenous Steroid
9	5	Munday MR (30)	1981	Clin Endocrinol	Correlations between Progesterone, Estradiol and Aldosterone Levels in the Premenstrual Syndrome
10	5	Epperson CN (31)	2002	Arch Gen Psychiat	Cortical Gamma-aminobutyric Acid Levels across the Menstrual Cycle in Healthy Women and those with Premenstrual Dysphoric Disorder: a Proton Magnetic Resonance Spectroscopy Study

### Analysis of Co-occurring Keywords and Burst Terms

**Figure 9:**
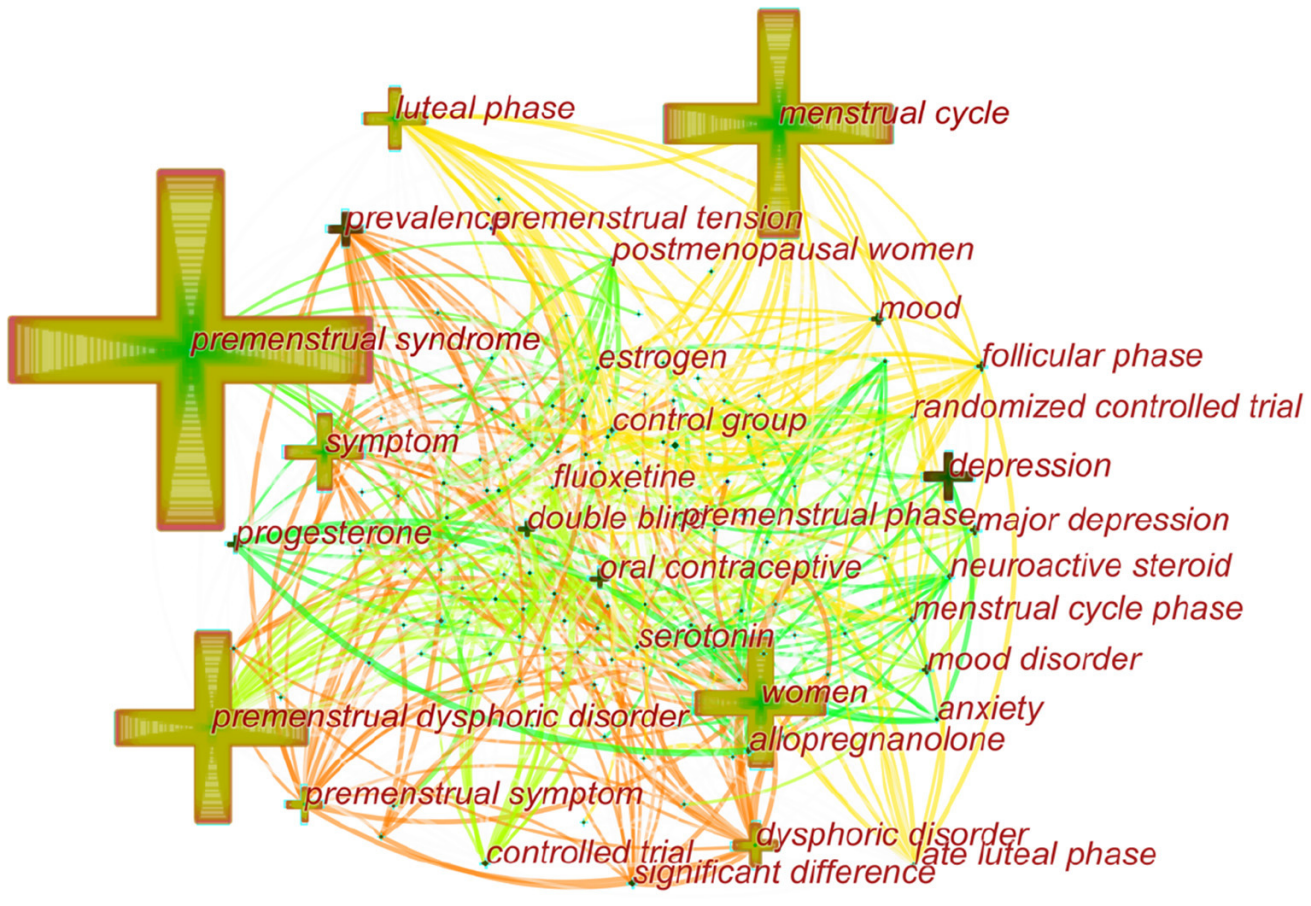
Map of keywords that published articles on PMS/PMDD during 1950–2018.

Over time, a knowledge map of keyword co-occurrence could reflect hot topics, and burst keywords (keywords that are cited frequently over a period of time) could indicate frontier topics. CiteSpace was utilized to construct a knowledge map of co-occurring keywords and identify the top 20 keywords in publications from 1945 to 2018 according to frequency, citation counts, and centrality (Table 6). Generating a keyword co-occurrence map resulted in 150 nodes and 842 links (Figure 9). Among the listed keywords, “premenstrual syndrome, menstrual cycle, premenstrual dysphoric disorder, women, symptom, luteal phase, depression, dysphoric disorder, premenstrual symptom, prevalence, double blind, progesterone, mood, oral contraceptive, and major depression” ranked ahead in both frequency and centrality, which suggested that they were the hotspots in the field. As we can see, “premenstrual syndrome and premenstrual dysphoric disorder” is ranked in the top three keywords, which is reasonable because they are our search terms. Except for them, other keywords actually reflect hotspots and topics that researchers are focusing on.

**Table 6:** Top 20 keywords in terms of frequency and centrality in PMS/PMDD research.

**Ranking**	**Freq**	**Keyword**	**Centrality**	**Keyword**
1	1556	Premenstrual syndrome	0.33	premenstrual syndrome
2	1024	Menstrual cycle	0.2	Symptom
3	845	Premenstrual dysphoric disorder	0.2	Premenstrual symptom
4	678	Women	0.14	Menstrual cycle
5	458	Symptom	0.12	Premenstrual dysphoric disorder
6	416	Luteal phase	0.12	Women
7	372	Depression	0.12	Dysphoric disorder
8	309	Dysphoric disorder	0.1	Luteal phase
9	306	Premenstrual symptom	0.1	Depression
10	276	Prevalence	0.08	Double blind
11	235	Double blind	0.08	Progesterone
12	223	Progesterone	0.06	Mood
13	193	Mood	0.06	Oral contraceptive
14	174	Oral contraceptive	0.06	Late luteal phase
15	162	Major depression	0.05	Prevalence
16	157	Follicular phase	0.05	Physical symptom
17	128	Estrogen	0.04	major depression
18	123	Significant difference	0.04	follicular phase
19	104	Late luteal phase	0.04	Estrogen
20	99	Controlled trial	0.04	Mood disorder

#### Research Hotspots and Topics of PMS/PMDD

Judging from the analysis of research hotspots, hot research topics revolve around the relationship of women's menstrual cycle with symptoms and progesterone. PMS/PMDD occurs only in women. According to the DSM-5, women with PMDD must have at least five predominantly affective symptoms with functional impairment, of which affective symptoms make up the largest proportion, such as mood swings, irritability, anger, and depressed mood. Therefore, the relationship between PMS/PMDD and the menstrual cycle has become a research hotspot, many studies have explored this area (9, 32, 33). Besides, PMDD subtypes research represents one of the hotspots (34). For instance, Chinese scholars proved that liver-qi invasion syndrome and liver-qi depression syndrome are the major subtypes of PMS/PMDD with epidemiological research (35, 36). Regarding cyclic mood disorders, cognitive, sensory, and emotional changes are linked to the menstrual cycle. The reason for this association is the ovarian hormones, especially the hormones progesterone and estrogen (37). Progesterone and its metabolites (e.g., isoallopregnanolone) have been recognized as hot topics in scientific research on PMS/PMDD (13, 38). Most studies confirmed that PMDD pathophysiology is rooted in impaired GABAA-R response to dynamic ALLO fluctuations across the menstrual cycle, manifesting in affective symptoms and poor regulation of physiologic stress response (39–41).

#### Research Frontiers of PMS/PMDD

**Figure 10:**
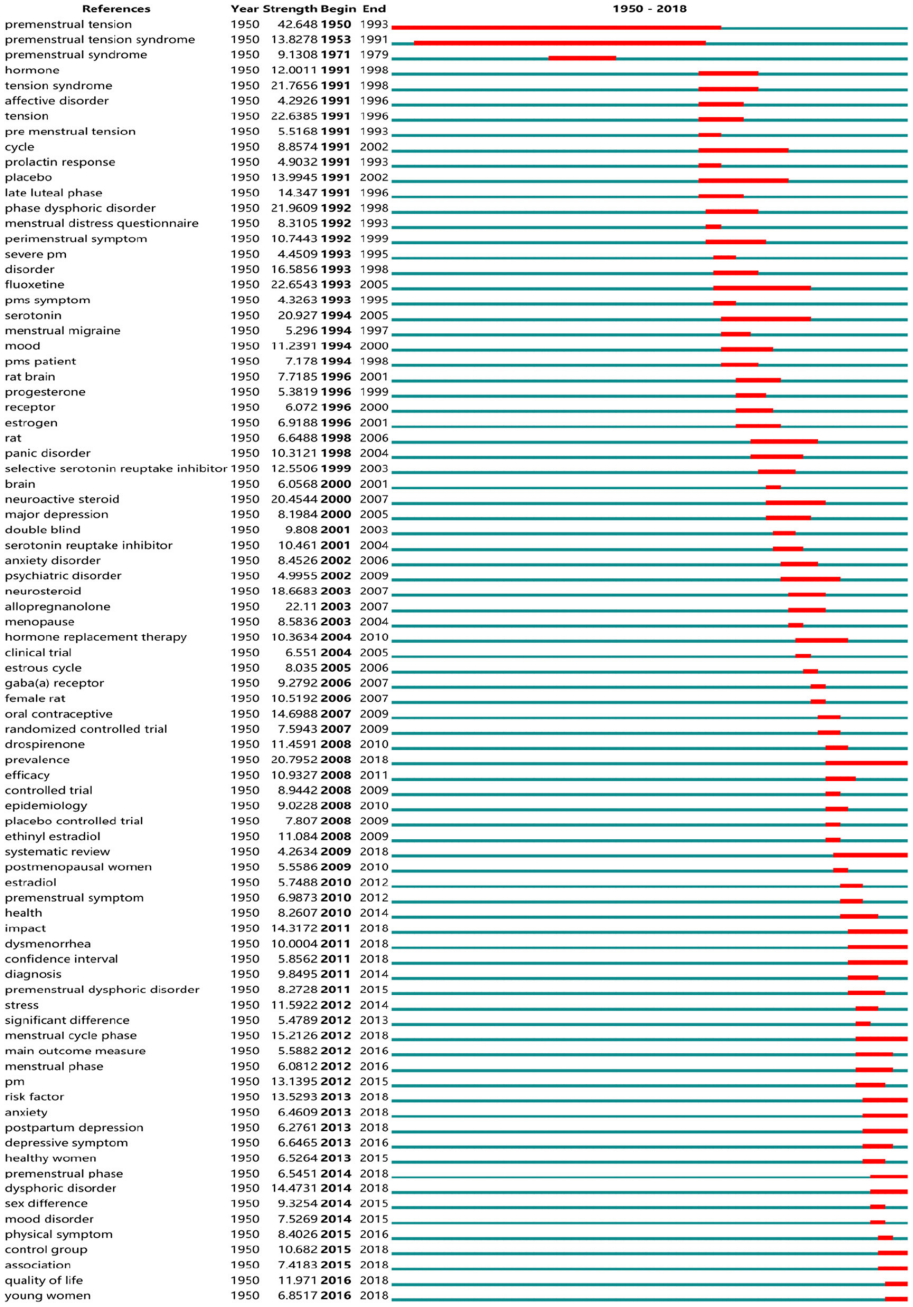
Top 84 keywords with the strongest citation bursts.

So-called “burst words” represent words that are cited frequently over a period of time. CiteSpace was used to detect burst keywords, which are deemed to be indicators of research frontier topics over time. In Figure 10, the time intervals are plotted on the green lines, while the periods of burst keywords are marked in red, indicating the beginning and end of the time interval of each burst. Among them, the keywords with citation bursts after 2008 were as follows: “prevalence” (2008–2018), “systematic review” (2009–2018), “impact” (2011–2018), “dysmenorrhea” (2011–2018), “confidence interval” (2011–2018), “menstrual cycle phase” (2012–2018), “risk factor” (2013–2018), “anxiety” (2013–2018), “postpartum depression” (2013–2018), “premenstrual phase” (2014–2018), “dysphoric disorder” (2014–2018), “control group” (2015–2018), “quality of life” (2016–2018), and “young women” (2016–2018).

**(1) Prevalence and impact in young women:**

Currently, it is estimated that 3–8% of women of reproductive age meet the strict criteria for PMDD (42). The assessment of published reports demonstrated that the prevalence of clinically relevant PMS/PMDD is probably higher due to the strict diagnostic criteria. Although DSM-IV or DSM-5 are the main diagnostic criteria for PMDD, we found an interesting phenomenon in which researchers used a wide variety of diagnostic tools to identify the incidence of the disease in searching for cold-related literature. There continue to be many problems in the implementation of diagnostic criteria (43). For one, patients need to report bothersome premenstrual symptoms, and clinicians should become more proficient in the diagnostic process to prevent the under diagnosis of these disorders (44). Estimating the prevalence of PMS/PMDD in greater scope and depth in different countries/regions is becoming increasingly important. In further studies, we found that young women are becoming the center of research, such as women in the Ukraine (45), in universities in Jordan (46), and in Japanese colleges (47), Israeli students (19), and other students all over the world. On the one hand, research output on the prevalence and impact of PMS/PMDD is also increasing around the world, such as consequences on female students' behavior, cognitive abilities, mental health status, and academic performance (48) and the impact of symptoms on the quality of life (49). However, researchers have not yet fully grasped the prevalence of the disease, lacking systematic reviews on prevalence.

**(2) Systematic Review evaluating risk factors:**

Systematic reviews typically are about a detailed and comprehensive plan and search strategy derived *a priori*, with the goal of synthesizing findings qualitatively or quantitatively (50). Systematic reviews of randomized controlled trials are fundamental to the practice of evidence-based medicine and to evaluate the effectiveness of drugs and methods for the treatment of uncomfortable symptoms. For example, cognitive-behavioral therapy (43), herbs (51), and vitamins and minerals are employed in the treatment of premenstrual syndrome (52). A systematic review of acupuncture and acupressure showed improvements in both the physical and psychological symptoms of PMS when compared to a sham control (53). A systematic review of treatment pointed out the curative effect of herbal remedies for the treatment of PMS (51, 52).

**(3) Association of anxiety and depression with menstrual cycle phases:**

Menstrual-related mood disorders such as PMDD are mood disorders related to the menstrual cycle. As major symptoms of many emotional disorders, anxiety and depression are the key directions of research. Moreover, some studies regarded PMS/PMDD as a risk factor for postpartum depression (54–56). Symptoms of irritability, emotional hypersensitivity, increased anxiety and food cravings, sleep difficulties, and decreased concentration characterize PMDD as well as depression, particularly atypical depression. A lifetime history of depression ranges from ~20 to 76% in samples of women diagnosed with PMS or PMDD (57). Recent studies have shown that menopause and menstrual cycle phases are time of intense hormonal fluctuation that can cause increased vulnerability to depression and anxiety (58). Besides, neuroimaging indicated that the emotional distress and dysregulation related to PMDD seem to be determined by structural, chemical, and functional brain signatures (59).

## Discussion

In this study, we have formulated research strategies to comprehensively analyze the current situation and development trend of PMDD research from 1945 to 2018 using a bibliometric analysis methodology. And we collected 2,833 papers for bibliographic records after evaluating 3,852 original results from the search for PMS/PMDD-related papers published in the Web of Science from 1945 to 2018 on the basis of the inclusion and exclusion criteria. Publications about PMS/PMDD could go back to 1950. In 1950, three articles were published: “Nephrotic syndrome with exaggerated premenstrual water and salt retention,” written by Lippman (60); “Premenstrual tension,” written by Morton (61), who worked at the New York Medical College; and “Premenstrual tension treated with vitamin A,” written by Argonz and Abinzano (62). Since then, PMDD research has gradually developed.

Our finding indicated that PMS/PMDD research is currently an area of active investigation and numerous findings are constantly emerging. Journals related to gynecology and endocrinology published most of PMS/PMDD research, such as Psychneuroendocrinology and Journal of Psychosomatic Obstetrics and Gynecology. USA had the largest number of publications, but China was the only country from the developing world to be in the top 10 countries that contributed PMS/PMDD research, showing its vast progress in life science over the past decade. And Research institutions were mainly in the USA (University of Rochester). Backstrom T ranked the first in more than 6,640 authors who contributed to the total number of publications, but Halbreich U leads the research direction.

In addition, we also find out the hotspots and trend of PMS/PMDD research through co-occurring keywords and burst terms, as described above. Prevalence and impact in young women, systematic review evaluating risk factors and association of anxiety and depression with menstrual cycle phases are research frontiers of PMS/PMDD. These forecasts have been confirmed in 2020–2021, such as research in Japanese college students (63) and systematic review on addictive behaviors across the menstrual cycle (64). But there are limitations in our research. Due to the large time span of submission of research papers, the statistical data of this paper is up to 2018. Even so, this study is enough to reflect the research hot spots and trends from 1945 to 2018.

## Conclusion

To the best of our understanding, this paper is the first bibliometric analysis of trends in PMS/PMDD research over the past decade. The data analysis process was relatively unbiased. The number of publications in PMS/PMDD research has been growing over the past decade. The USA, ENGLAND and SWEDEN were the top three countries contributing to PMS/PMDD studies. There was active collaboration between developed countries. China was the sole developing country that made it into the list of the top ten countries contributing to PMS/PMDD studies. The USA and its institutions still occupy the leading position. UNIV PENN, the NIMH, and UNIV CALIF LOS ANGELES may be ideal candidates for academic cooperation. Prevalence and impact in young women, systematic review evaluations of risk factors, and the association of anxiety and depression with menstrual cycle phases may be frontiers in this field, and researchers should follow closely relevant studies in the coming years.

## Data Availability Statement

The original contributions presented in the study are included in the article/supplementary material, further inquiries can be directed to the corresponding author.

## Author Contributions

MG conceived and designed the studies and drafted the manuscript. MG, HS, and DG performed the experiments, analyzed the data, contributed materials/analysis tools, prepared figures and/or tables, and approved the final draft. XC, LA, and MQ provide critical services. Fund support was supplied by DG and MQ. All authors read and approved the final manuscript.

## Conflict of Interest

The authors declare that the research was conducted in the absence of any commercial or financial relationships that could be construed as a potential conflict of interest.

## Abbreviations

PMS: premenstrual syndrome
PMDD: premenstrual dysphoric disorder
WoSCC: Web of Science Core Collection
DSM-5, Diagnostic and Statistical Manual of Mental Disorders: Fifth Edition.
